# *miR-200b*, *ZEB2* and *PTPN13* Are Downregulated in Colorectal Carcinoma with Serosal Invasion

**DOI:** 10.3390/biomedicines10092149

**Published:** 2022-09-01

**Authors:** Branislava Ranković, Emanuela Boštjančič, Nina Zidar, Margareta Žlajpah, Jera Jeruc

**Affiliations:** Faculty of Medicine, Institute of Pathology, University of Ljubljana, Korytkova 2, 1000 Ljubljana, Slovenia

**Keywords:** colorectal carcinoma, serosal invasion, T stage, epithelial-mesenchymal transition, miR-200 family, target genes, ONECUT2, PTPN13

## Abstract

Serosal invasion is an independent negative prognostic factor in certain cancers, including CRC. However, the mechanisms behind serosal invasion are poorly understood. We therefore assumed that epithelial-mesenchymal transition (EMT) might be involved. Our study included 34 patients with CRC, 3 stage pT2, 14 stage pT3 and 17 showing serosal invasion (stage pT4a according to TNM staging system). RNA isolated from formalin-fixed paraffin-embedded tissue samples was analysed for expression of the *miR-200* family and their target genes *CDKN1B, ONECUT2, PTPN13, RND3, SOX2, TGFB2* and *ZEB2* using real-time PCR. We found upregulation of *miR-200b* and *ONECUT2* in CRC pT3 and pT4a compared to normal mucosa, and downregulation of *CDKN1B* in CRC pT3. Moreover, we observed, downregulation of *miR-200b, PTPN13* and *ZEB2* in CRC with serosal invasion (pT4a) compared to pT3. Our results suggest the involvement of partial EMT in serosal invasion of CRC. In addition, *PTPN13* seems to be one of the important regulators involved in serosal invasion, and *ONECUT2* in tumour growth.

## 1. Introduction

Colorectal adenocarcinoma (CRC) is one of the most common cancers and the second leading cause of cancer-related deaths in Europe. Its burden is expected to increase as the population ages [1]. Based on the American Joint Committee on Cancer (AJCC) tumour–node–metastasis (TNM) staging system [2] for CRC, in patients who are negative for distant metastases, the stage depends on the degree of invasion into the intestinal wall (pT stage 1–4) and the number of regional lymph nodes positive for metastases [3]. The final substage of the invasion in the intestinal wall is peritoneal involvement or serosal invasion and is labelled stage pT4. It is an independent prognostic factor in CRC and is associated with increased risk of intraperitoneal spread, recurrence and decreased overall survival [4,5,6,7,8]. While several studies have investigated mechanisms leading to peritoneal carcinomatosis [9,10], serosal invasion is still poorly understood. Due to its negative impact on prognosis, it is important to understand the mechanisms or regulators of direct cancer invasion of the serosa. Several pathogenetic mechanisms, including epithelial-mesenchymal transition (EMT), could be involved.

EMT is a process by which immotile epithelial cells undergo a deep molecular reprogramming, gradually losing their epithelial features and acquiring a migratory and invasive mesenchymal phenotype [11,12,13]. Despite extensive research, the exact role of EMT in CRC remains controversial. Previous studies have indicated that EMT plays an important role in CRC, in both its development and progression [14,15,16,17,18]. Furthermore, EMT is believed to be one of the key processes in the development of metastases in CRC [17,19,20,21]. Our past results revealed that EMT plays a role only in its partial “form” in the development, progression and metastasis of CRC [16,17]. However, there are limited data on the involvement of EMT in invasion through different layers of the bowel wall and finally through the peritoneal membranes, i.e., serosal invasion.

The aim of our study was to investigate the involvement of EMT in local progression of CRC with an emphasis on serosal invasion. Our approach is based on the results of our previous studies showing that downregulation of all five members of the *miR-200* family is a key feature and one of the most useful molecular markers of EMT [16,17,22]. We therefore analysed the expression of the *miR-200* family and their target genes in CRC in low pT stages (pT2-3) and compared it with advanced CRC, showing serosal invasion (pT4a).

## 2. Materials and Methods

### 2.1. Tissue Samples

Tissue samples from 34 patients with CRC were included in the study. Patients treated by radiotherapy, chemotherapy or biologic drugs prior to surgery were excluded from the study. Samples of CRC and corresponding normal mucosa were collected retrospectively from the archives of the Institute of Pathology, Faculty of Medicine, University of Ljubljana. Tissue samples were fixed in 10% buffered formalin and embedded in paraffin (FFPE) for the purpose of routine histopathologic examination. On the basis of histopathological features, the samples were divided into two groups: patients without serosal invasion (CRC pT2-T3) and patients with serosal invasion (CRC pT4a). Serosal invasion was defined according to the International Collaboration on Cancer Reporting (ICCR), as a tumour infiltrating the bowel wall and breaching the visceral peritoneum, with tumour cells either visible on the visceral peritoneal surface or separated from the peritoneal surface by inflammatory cells only. A peritoneal or omental deposit that was discontinuous from the primary mass was defined as metastatic disease (M1c) and therefore not included in the study [23,24].

### 2.2. RNA Isolation from FFPE Tissue Slides

Tissue samples were cut at 10 μm from FFPE tissue blocks, andfour 10 μm sections were used for the isolation procedure. For cases with peritoneal involvement (pT4a), the representative tissue block containing clear peritoneal invasion was selected.

AllPrep DNA/RNA FFPE kit (Qiagen, Hilden, Germany) was used for total RNA isolation according to the manufacturer’s instructions. Concentration was measured using the spectrophotometer ND-1000 (Nanodrop, Thermo Fisher Scientific, Waltham, MA, USA) at the wavelengths 260, 280 and 230 nm.

Reverse transcription (as described under Section 2.4.1) followed by amplification of the *GAPDH*, a housekeeping gene (amplicon length is 100 bp), using qPCR and Sybr Green technology, was performed as quality control. All of the samples included in the current study had passed amplification of *GAPDH*, and those that did not amplify were not included in the study.

### 2.3. Expression of microRNAs Family miR-200 Using qPCR

Expression of *miR-200* was analysed using TaqMan probes (Thermo Fisher). Prior to qPCR, two separated pools of RNA samples were created. One pool was obtained from tumour samples and the other was obtained from corresponding normal mucosa. After reverse transcription, the cDNA was diluted from 4-fold dilution to 1024-fold dilution (5 steps), and the probes were tested for qPCR efficiency. All the reactions for the efficiency of qPCR for each of the specific probe were performed in triplicate on RotorGene Q (Qiagen, Hilden, Germany).

#### 2.3.1. Reverse Transcription (RT) for Quantification of miRNAs

MicroRNA TaqMan RT kit (Applied Biosystems, Foster City, CA, USA) and looped primers for specific RT of miRNAs were utilized following the manufacturer’s protocol. *miR-141, miR-200a, miR-200b, miR-200c* and *miR-429* were analysed relative to the geometric mean of expression of *RNU6B* and *miR-1247b*. Briefly, RT reaction master mix contained 10 ng of total RNA sample, 1.0 μL of MultiScribe Reverse Transcriptase (50 U/μL), 1.0 μL of Reverse Transcription Buffer (10×), 0.1 μL of dNTP (100 mM), 0.19 μL RNAase inhibitor (20 U/μL), and 2.0 μL of RT primer (5×) in the final volume of 10 μL. The reaction conditions were as follows: at 16 °C for 30 min, at 42 °C for 30 min and at 85 °C for 5 min.

#### 2.3.2. Quantitative Real-Time PCR (qPCR) of miRNAs

miRNAs expression was assessed in 10 μL qPCR master mix, containing 5.0 μL TaqMan 2x FastStart Essential DNA Probe Master (Roche, Basel, Switzerland), 0.5 μL TaqMan assay and 4.5 μL RT products diluted 100-fold. All qPCR reactions were performed in duplicates as follows: initial denaturation at 95 °C for 10 min, and 40 cycles for 15 s at 95 °C (denaturation) and for 60 s at 60 °C (primers annealing and elongation). qPCR was performed using RotorGene Q (Qiagen, Hilden, Germany) with signal collected at the endpoint of every cycle.

### 2.4. Expression of Target Genes of miR-200 Family Using qPCR

qPCR was used for the analysis of mRNA expression of protein-coding genes. A pre-designed mixture of probes and primers based on the TaqMan methodology (Thermo Fisher) was used for target mRNA expression. Accordingly, for selected genes, we chose TaqMan primers and probes that amplify and detect PCR products less than 100 bp long. Again, the same two pools of RNA as described above (Section 2.3), obtained from normal mucosa and from CRC, were used to test efficiency. After RT (Section 2.4.1) and PreAmp (Section 2.4.2), the pre-amplified cDNA was diluted, ranging from 5-fold dilution to 625-fold dilution (4 steps), and all of the used probes were tested for qPCR efficiency. All the reactions were performed in triplicate on RotorGene Q (Qiagen, Hilden, Germany). The mRNAs *CDKN1B*, *ONECUT2*, *PTPN13*, *RND3*, *SOX2*, *TGFB2* and *ZEB2*, which are proven targets of the miR-200 family in CRC and were expressed in our previous study (Rankovic, 2019), were analysed relatively to the geometric mean of IPO8 and B2M.

#### 2.4.1. Reverse Transcription (RT) for Quantification of mRNAs

Total RNA was reverse-transcribed using random primers and OneTaq RT-PCR Kit (New England Biolabs, Ipswich, MA, USA) according to the manufacturer’s protocol. For RT reactions, 60 ng of total RNA (3 μL) was first incubated with 1.0 µL of Random Primer Mix at 70 °C for 5 min. Further, to obtain 10 μL of RT reaction, 5.0 μL of M-MuLV Reaction Mix and 1.0 μL of M-MuLV RTase were added to 4.0 μL of reaction mix after random priming. RT reaction was performed as follows: at 25 °C for 5 min, at 42 °C for 60 min and at 80 °C for 4 min.

#### 2.4.2. Pre-Amplification of mRNAs

TaqMan PreAmp Master Mix (Applied Biosystems, Foster City, CA, USA) was used following RT to pre-amplify the resulting cDNA. A 10 µL PreAmp reaction mix contained a pool of 0.2× diluted probes (2.5 µL), PreAmp Master mix 2× (5.0 µL) and cDNA (2.5 µL), according to the manufacturer’s protocol. The reaction conditions were as follows: 10 min at 95 °C and 10 cycles of 15 s at 95 °C and 4 min at 60 °C.

#### 2.4.3. Quantitative Real-Time PCR (qPCR) of mRNAs

The resulting PreAmp reaction was diluted 5-fold. The 10 μL reaction volume of qPCR contained 5.0 μL of 2× FastStart Essential DNA Probe Master Mix (Roche, Basel, Switzerland) and TaqMan probe, 0.5 μL of specific TaqMan probe and 4.5 μL of diluted PreAmp reaction. Thermal conditions were as follows: 50 °C for 2 min, initial denaturation at 95 °C for 10 min and 40 cycles of denaturation at 95 °C for 15 s and annealing at 60 °C for 1 min. All qPCR analyses were performed in duplicates on Rotor Gene Q (Qiagen, Hilden, Germany). The signal was collected at the endpoint of each cycle.

### 2.5. Statistical Analysis

First, all Cqs were corrected for PCR efficiencies. The results were presented as relative gene expression. The Cq of the gene of interest (GOI, Ct_GOI_) was calculated relative to the geometric mean of the Cqs of reference genes (RGs, Ct_RG_), and named ΔCq. Statistical analysis of data was performed using SPSS version 27 (SPSS Inc., Chicago, IL, USA). All analysed differences were considered significant at *p* < 0.05. The Wilcoxon rank test and ΔCq were used to calculate statistically significant expression differences of mRNAs and miRNAs between tumours and adjacent normal tissue. For statistical comparison of relative expression of mRNA and miRNA between independent groups of samples, the Mann-Whitney test was used with either ΔCq or ΔΔCq. The Spearman correlation coefficient was calculated to determine whether there was any association between status T and expression of selected miRNAs and mRNAs.

## 3. Results

### 3.1. Patients and Tissue Samples

**Figure 1:**
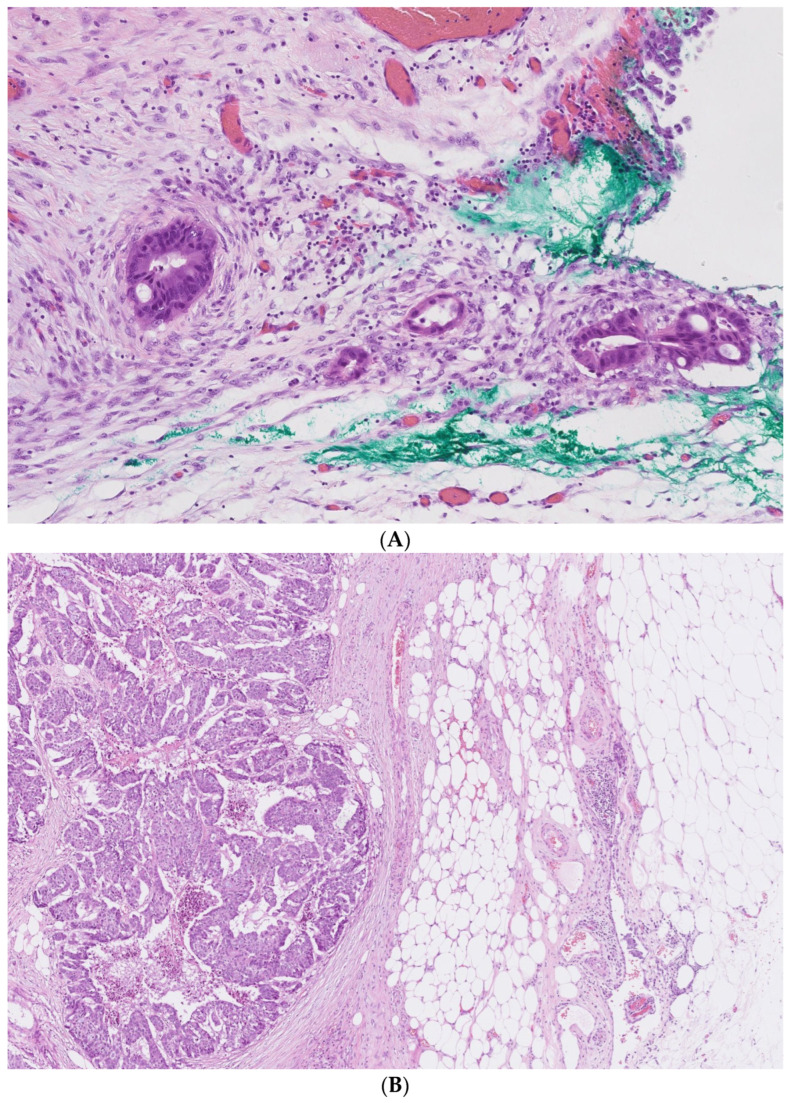
Serosal invasion in carcinoma (pT4a) (**A**) and carcinoma showing invasion into pericolic connective tissues without serosal invasion (pT3) (**B**).

There were 34 patients with different stages of CRC included in the study. The pTNM stage was determined after resection according to international guidelines [25]. In 16 patients, serosal invasion was confirmed histologically (Figure 1). The clinicopathologic characteristics of the patients are presented in Table 1.

### 3.2. Expression of miR-200 Family and Its Target Genes in Carcinoma with and without Serosal Invasion Compared to Normal Mucosa

**Figure 2:**
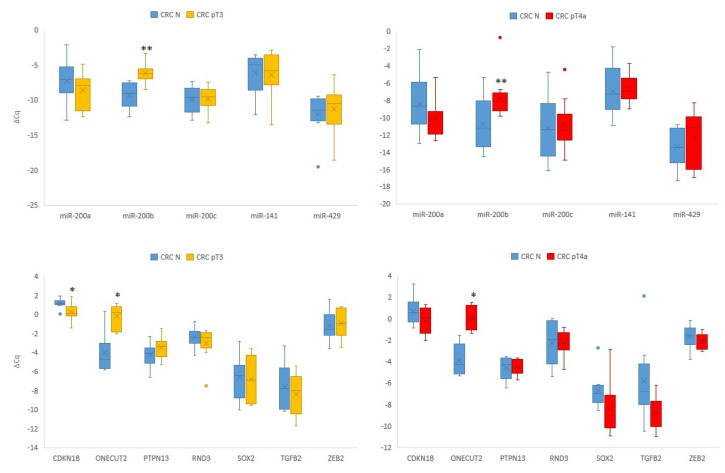
Expression of the *miR-200* family and their target genes in CRC pT3 and CRC pT4a in comparison to corresponding normal mucosa. Abbreviations: CRC, colorectal carcinoma; ΔCq, delta cycle of quantitation; N, normal mucosa adjunct to CRC; pT3, pathological stage T3; pT4a, pathological stage T4a according to pathologic tumour–node–metastasis classification [25]; *, *p* < 0.05; **, *p* < 0.01.

We observed significant upregulation of *miR-200b* (*p* = 0.003) and *ONECUT2* (*p* = 0.018) in the tumour tissue of CRC pT3 compared to the corresponding normal mucosa, as well as downregulation of *CDKN1B* (*p* = 0.013). Similarly, we observed significant upregulation of *miR-200b* (*p* = 0.002) and *ONECUT2* (*p* = 0.028) in CRC pT4a tumour tissue with serosal invasion compared to the corresponding normal mucosa. The results are summarized in Figure 2. No other miRNA or mRNA was differentially expressed.

We further compared the expression of the analysed transcripts in all normal mucosa samples (n = 34) with CRC T3 (n = 15) and CRC pT4a (n = 16). Due to the small number of patients with CRC pT2 (n = 3), we did not perform statistical analysis in those samples since it would have given us unreliable results. We observed statistically significant upregulation for *miR-200b* (*p* < 0.001)*, miR-429* (*p* = 0.021)*, ONECUT2* (*p* < 0.001) and *PTPN13* (*p* = 0.030) in CRC pT3. Comparing all normal samples to CRC pT4a, upregulation of *ONECUT2* (*p* < 0.001) and downregulation of *miR-200a* (*p* = 0.005)*, CDKN1B* (*p* = 0.028) and *SOX2* (*p* = 0.022) were observed.

These data suggest that *miR-200b* and *ONECUT2* are important contributors in local CRC spread.

### 3.3. Comparison of Expression of miRNAs and Its Target Genes in Carcinoma with Serosal Invasion Compared to Carcinoma without Serosal Invasion

**Figure 3:**
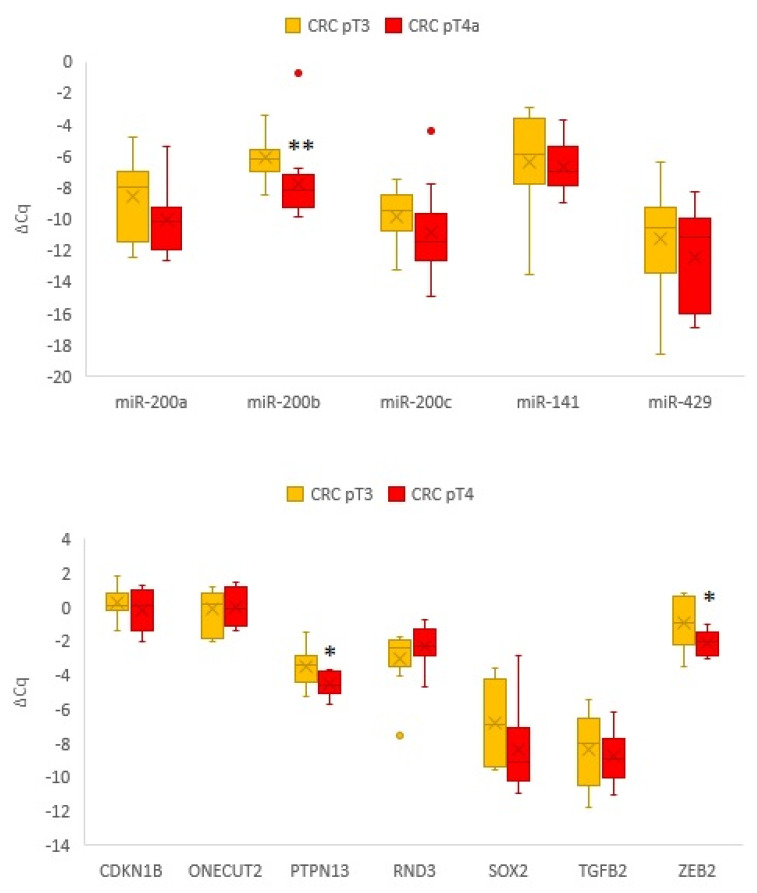
Expression of the *miR-200* family and their target genes in CRC pT4a in comparison to CRC pT3. Abbreviations: CRC, colorectal carcinoma; ΔCq, delta cycle of quantitation; pT3 and pT4a, pathological stage T3 and T4a, respectively, according to pathologic tumour–node–metastasis classification [25]; *, *p* < 0.05; **, *p* < 0.01.

To evaluate the contribution of EMT in the invasion of cancer cells through serosal membranes, we further compared the expression of the analysed transcripts between CRC pT3 and CRC pT4a. We observed all members of the *miR-200* family to be downregulated in pT4a; however, only *miR-200b* reached statistically significant downregulation (*p* = 0.003). Furthermore, we also observed significant downregulation for *PTPN13* (*p* = 0.037) and *ZEB2* (*p* = 0.017) in CRC pT4a compared to CRC pT3. The results are summarized in Figure 3.

### 3.4. Correlations between miRNAs, Their Target Genes and Status T

Association analysis showed a correlation between the increasing T stage (including adjacent normal mucosa as T0) and the expression of *CDKN1B, ONECUT2, SOX2* and *TGFB2* (Rho = −0.488, *p* = 0.002; Rho = 0.732, *p* < 0.001; Rho = −0.346, *p* = 0.049; Rho = −0.394, *p* = 0.031, respectively), as well as with the expression of *miR-200a, miR-200b* and *miR-429* (Rho = −0.319, *p* = 0.008; Rho = 0.388, *p* = 0.001; Rho = 0.273, *p* = 0.033, respectively). Comparing only tumour samples without the corresponding normal mucosa, an inverse association of increasing T stage with the expression of *PTPN13* and *ZEB2* (Rho = −0.619, *p* = 0.014; Rho = −0.651, *p* = 0.006; respectively) as well as with the expression of *miR-200a, miR-200b* and *miR-200c* (Rho = −0.447, *p* = 0.015; Rho = −0.426, *p* = 0.024; Rho = −0.433, *p* = 0.021, respectively) was found. The only observed correlation between miRNAs and their target gene expression was between *miR-429* and *ZEB2* (Rho = 0.655, *p* < 0.001).

## 4. Discussion

Serosal invasion in CRC is one of the well-recognized adverse prognostic features associated with an increased risk for tumour spread and recurrence; however, its mechanisms are still not completely elucidated. In the present study, we analysed the expression of the *miR-200* family and their target genes to investigate the possible role of EMT in local tumour spread through different layers of the bowel wall, focusing on serosal invasion. Our previous studies support the hypothesis that partial EMT is induced during the progression from normal colon mucosa to adenoma and carcinoma and that partial MET is involved in CRC metastases (12,13). We therefore hypothesized that partial EMT might also be involved in local tumour spread. There are two main findings in the present study. First, we observed downregulation of *miR-200b*, *PTPN13* and *ZEB2* in CRC with serosal invasion (pT4a) compared to pT3 tumours. Second, we observed upregulation of *miR-200b* and *ONECUT2* in CRC compared to normal mucosa in both pT3 and pT4a CRC, downregulation of *CDKN1B* in CRC pT3 and upregulation of ONECUT2 with increasing status T.

Expression of *miR-200b* was downregulated in CRC with serosal invasion compared to locally less advanced CRC. Indeed, several studies have shown that downregulation of *miR-200b* is associated with the progression of CRC. In a large series of 1141 CRC cases, Slattey et al. found *miR-200b* to be decreased in advanced stages of CRC [26]. The expression of *miR-200b* was significantly downregulated at the invasive front of tumours compared to normal and core tumour tissues [14,15,17], suggesting that downregulation of *miR-200b* and the subsequent upregulation of their target genes promotes a switch in cell phenotype, resulting in the increased invasiveness and metastatic potential of tumour cells.

However, *miR-200b* was upregulated in all the tumour samples investigated compared to normal mucosa. Similar to our results, Diaz et al. and Pan et al. demonstrated *miR-200b* upregulation in tumour tissue versus normal adjacent tissue [27,28]. It has been shown that *miR-200b* stimulates tumour growth by targeting and negatively regulating CDKN1B, a negative regulator of the cell cycle, in TGFBR2-null CRC cell lines [29]. This is in accordance with our study that showed downregulation of *CDKN1B* in CRC compared to normal mucosa. Fu et al. showed that both *CDKN1B* and *RND3* are the direct targets of *miR-200b*. While *miR-200b-*induced *CDKN1B* degradation promotes cancer cell proliferation, reduced expression of *miR-200b* was accompanied by an increased expression of *RND3*, an important regulator of cell migration [29,30]. It is possible that during the early stages of carcinogenesis, upregulation of *miR-200b* has a role in proliferation by inhibiting *CDKN1B*, while later, during the progression of CRC from pT3 to pT4a, downregulation of *miR-200b* promotes invasiveness and migration, i.e., serosal membrane invasion by targeting other yet unidentified genes. It has been shown that in addition to EMT, *miR-200b* can also regulate tumour proliferation, apoptosis, invasion, migration and chemotherapy resistance through the WNT/β-catenin pathway, MAPK signalling pathway, PI3K/Akt pathway and others [31].

We observed downregulation of *PTPN13* and *ZEB2* in CRC with serosal invasion (pT4a) compared to CRC pT3. *ZEB2*, a target of all miRNAs of the *miR-200* family, is an EMT-inducing transcription factor, previously shown to be associated with the progression of CRC and the risk of distant but not local recurrence [32,33]. Our previous study showed *ZEB2* to be downregulated in cancers with nodal metastases compared with those without [17]. Our present study could not confirm the involvement of *ZEB2* in local CRC spreading, including serosal invasion. Therefore, other EMT transcription factors might be involved in the progression of CRC. The answer might be in downregulation of *PTPN13*.

Our present study showed upregulation of *PTPN13* in CRC; however, it was lower in samples with serosal invasion (pT4a) than in those without (pT3). *PTPN13* (also called FAP-1) is a non-receptor protein tyrosine phosphatase that acts in a number of important growth and apoptosis pathways [34]. *PTPN13* negatively regulates EMT by inhibiting Slug and Snail, two main EMT transcription factors and also through cell junction stabilization via its positive role in desmosome formation [35,36]. Regarding breast cancer animal model and cell lines, Hamyeh et al. suggested that *PTPN13* inhibits tumour aggressiveness through a SNAIL- and ZEB-independent MET-like transition and cell junction stabilization [36]. Our results thus indicate a tumour suppressor role of *PTPN13* in the early stages of CRC growth, while in serosal invasion it may act as a tumour promoter via activation of Slug and Snail and inhibition of FAS-induced apoptosis [37,38,39].

Compared with healthy tissues, *PTPN13* expression is decreased in different types of cancer [34]. Accordingly, its expression is linked to less aggressive tumours and better patient survival [34]. Similar to our results, a recent study on the diagnostic and prognostic role of PTPN family members in digestive tract cancers using Oncomine, Ualcan and TCGA data found the expression of *PTPN13* in CRC to be upregulated compared to normal samples. The expression did not correlate with TNM stage and overall survival [40]. We found no correlation between *PTPN13* and its regulator *miR-200c*. In addition to post-transcriptional regulation of *PTPN13* in CRC by miRNAs [41,42], its expression might be regulated through promoter hypermethylation or loss of heterozygosity [43,44,45,46].

We observed another interesting result. Although not differentially expressed between pT3 and pT4a, expression of *ONECUT2* increased with the pT stage and might be thus indirectly related to local tumour spread. *ONECUT2* is a relatively new member of the *ONECUT* transcription factor family that can widely regulate protein expression associated with cell proliferation, migration, adhesion, differentiation and cell material metabolism [47]. The role of *ONECUT2* in cancer progression has been found to be elevated in different types of cancer, including CRC [16,48,49,50,51,52]. Chen et al. showed that the expression of *ONECUT2* correlated with poor prognosis in patients with gastric cancer. In addition, similar to our results in CRC, they noted a correlation of *ONECUT2* with tumour stage [50]. In contrast, levels of *ONECUT2* were found to be lower in breast cancer, and patients with low expression levels showed worse survival than those with high expression levels of *ONECUT2* [53]. The studies involving *ONECUT2* expression and its regulation in CRC are limited. Using CRC cell lines and tumour tissues, Sun et al. have shown that *miR-429* inhibits cell growth and invasion by targeting *ONECUT2* [52]. However, we were not able to confirm a correlation between *miR-429* and *ONECUT2* expression. Recent studies described novel ONECUT2 regulatory mechanisms. In CRC, circular RNA (circ_0084615) serves as a sponge for miR-599, which directly binds to ONECUT2 [54]. Furthermore, studies on CRC cells showed that by silencing circ_0084615, EMT-related markers ZEB2 and vimentin are reduced and E-cadherin is elevated. It has been suggested that in addition to miRNA and circRNAs, the expression of *ONECUT2* can be regulated by DNA hypermethylation [55] and even hypomehylation [56]. Recently, a single-nucleotide polymorphism in *ONECUT2* gene was identified as strong predictive marker for CRC risk [57]. However, while being upregulated in all stages of the cancer progression through the bowel wall, *ONECUT2* does not seem to contribute significantly to the invasion through the serosal membranes, but rather is responsible for the continuous growth of tumours.

There are several advantages and limitations of our study. As the purpose of the study was to compare tumours in different pT stages, we used the whole tissue sections of the resected tumours. In samples with serosal invasion, in addition to EMT, mesothelial-to-mesenchymal transition contributes to gene expression results [58]. This limitation could be resolved using laser-captured microdissection to include only representative tumour cells in the analysis. Another limitation of our study is the small number of patients, particularly CRC pT2. Nucleic acids from FFPE tissue blocks are difficult to analyse, and specialized methodologies are often necessary. One of the reasons for the relatively small sample size is that after initial RNA isolation, only samples that passed the quality control were included in the study. Finally, we analysed only two EMT transcription factors, *ZEB1* and *ZEB2*, *ZEB1* being below detection limit or even not expressed. However, although the current study has several limitations, there are limited data on molecular analyses in CRC with serosal invasion compared to those without. We therefore believe that *PTPN13, ZEB2* and *ONECUT2* might be promising targets for further research.

## 5. Conclusions

Our results suggest that decreased expression of *miR-200b, ZEB2* and *PTPN13* is involved in local tumour spread of CRC, particularly in serosal invasion (pT4a). We also observed that increasing expression of *ONECUT2* is associated with tumour growth and its spread through the bowel wall. Our findings further support that at least certain components of EMT might be involved in serosal invasion in CRC.

## Figures and Tables

**Table 1:** Clinicopathologic characteristics of the patients included in the study.

T Stage ^1^	Age (Mean ± SD)	Gender (M:F)	Location (RC/LC/RSC)
**pT2 (n = 3)**	68 ± 5.88	1:2	1/0/2
**pT3 (n = 14)**	76.5 ± 11.10	6:8	6/1/7
**pT4a (n = 17)**	69.5 ± 14.4	13:4	5/3/9

^1^ According to pathologic tumour–node–metastasis classification [25]. Abbreviations: RC, right colon; LC, left colon; RSC, rectosigmoid colon.

## Data Availability

The datasets used and analysed during the current study are available from the corresponding author on reasonable request.
